# Personality Predictors of Successful Development: Toddler Temperament and Adolescent Personality Traits Predict Well-Being and Career Stability in Middle Adulthood

**DOI:** 10.1371/journal.pone.0126032

**Published:** 2015-04-28

**Authors:** Marek Blatný, Katarína Millová, Martin Jelínek, Terezie Osecká

**Affiliations:** 1 Department of Personality and Social Psychology, Institute of Psychology, The Czech Academy of Sciences, Brno, Czech Republic; 2 Department of Research Methodology, Institute of Psychology, The Czech Academy of Sciences, Brno, Czech Republic; Georgia State University, UNITED STATES

## Abstract

The aim of the study was to predict both adaptive psychological functioning (well-being) and adaptive social functioning (career stability) in middle adulthood based on behaviors observed in toddlerhood and personality traits measured in adolescence. 83 people participated in an ongoing longitudinal study started in 1961 (58% women). Based on children’s behavior in toddlerhood, three temperamental dimensions were identified – positive affectivity, negative affectivity and disinhibition. In adolescence, extraversion and neuroticism were measured at the age of 16 years. Various aspects of well-being were used as indicators of adaptive psychological functioning in adulthood: life satisfaction, self-esteem and self-efficacy. Career stability was used as an indicator of adaptive social functioning. Job careers of respondents were characterized as stable, unstable or changeable. Extraversion measured at the age of 16 proved to be the best predictor of well-being indicators; in case of self-efficacy it was also childhood disinhibition. Extraversion in adolescence, childhood disinhibition and negative affectivity predicted career stability. Findings are discussed in the context of a theoretical framework of higher order factors of the Big Five personality constructs, stability and plasticity.

## Introduction

The past almost 100 years of personality research has yielded ample evidence that personality dispositions significantly co-determine human life. Based on personality traits we are able, to a certain extent, to predict how people will experience and behave in certain life domains. One of the most studied areas are life satisfaction and well-being, where it has been established that personality traits contribute significantly to how people evaluate their lives [1–4]. But personality traits play an important role in other areas of human life as well—they influence academic achievement [5], occupational choice [6], stress resistance and selection of coping strategies [7], likelihood of burnout [8], partner choice [9] and even fertility and number of children [10]. We can therefore say that personality traits largely determine the future course of life. One of the most suitable frameworks for the study of how traits influence human life is life-span psychology and its topic of successful development. This study builds on recent studies that have considered the relations between personality and psychological functioning, well-being or the relations between personality and social functioning. It strives to enrich it in two ways—in prediction it focuses on both components of successful development and takes toddler temperament into account.

### Life-span psychology and successful development

Lifespan psychology is a field of developmental psychology that deals with human development from conception to death [11]. The first comprehensive theories attempting to explain the evolution of man were formulated at the beginning of the 20^th^ century [12, 13], but development throughout life had not been systematically described before the mid-20^th^ century [14]. One of the areas of life-span psychology is successful development, which has received more attention in recent years [15]. Successful development is associated with maintaining a balance in different levels of psychological and social functioning [16, 17], with good health [18] or effective social functioning [19]. Paul B. Baltes defined successful development as maximizing gains and minimizing losses [20].

Current empirical research usually distinguishes between two aspects of successful development. It is approached primarily in terms of external (objective, social) and internal (subjective, psychological) criteria. External criteria relate in particular to adaptation to social norms and to comparisons with social requirements and are dependent on culture [13, 20, 21]. Internal criteria relate mainly to well-being and comparisons with own expectations and the ideal self [22, 23].

Both components of adaptive functioning are highly interconnected [24–26]. The professional domain, for instance, plays a significant role in human well-being: as research findings have shown, unemployment tends to considerably decrease its level [27]. At the same time, occupational status and job satisfaction count among important predictors of well-being [28]. In addition, higher education might enhance personality coherence [29]. Other aspects of social functioning that seem to have a profound impact on well-being include quality of attachments, marriage/partnership satisfaction [30, 31] and socioeconomic status [32, 33].

### Personality and adaptive psychological functioning: well-being

In recent decades, well-being has become one of the most widely studied areas of psychological research [34, 35]. During that period, a series of theoretical concepts of well-being were created, of which two assumed a significant position among researchers—subjective well-being (SWB) [36, 37] and psychological well-being (PWB) [38, 39]. SWB is based on the hedonic tradition [40] and it involves a high level of experienced positive emotions, low level of experienced negative emotions and cognitive evaluation of one's own life as a whole—life satisfaction [36]. PWB is based on the eudaimonic tradition [41] and it is considered to be the result of the implementation of positive life pursuits such as autonomy, personal growth, environmental mastery and purpose in life [38, 42].

Previous research has studied mainly SWB to identify variables or factors that are associated with well-being. Only modest connections were found between well-being and situational factors [43], whereas research has shown significant and mainly stable relations between well-being and personality variables [4].

SWB is primarily associated with extraversion and neuroticism [1, 44]. With the establishing of the five-factor model of personality, the research has extended to other personality traits—conscientiousness, agreeableness and openness to experience [45]. In addition to extraversion and neuroticism, conscientiousness and agreeableness turned out to be other significant correlates [46]. The results of these studies were summarized in their meta-analyses by DeNeve and Cooper [47] and Steel, Schmidt, and Schulz [48]. DeNeve and Cooper [47] found correlations between SWB and personality traits in the expected direction—positive with extraversion, conscientiousness, agreeableness and openness to experience, and negative with neuroticism—but not in the expected size, as suggested by previous research: personality traits explained only 4% of the variance of the indices of SWB. This meta-analysis was followed up by Steel, Schmidt, and Schulz [48], who took into account the effect of commensurability, i.e. the construct variation in personality and construct variation in SWB, and they used the multivariate approach. According to their findings, the relations between well-being and personality are much stronger; SWB total variance accounted for by personality reached as high as 39% or 63%, disattenuated.

Stable relations between personality and well-being were proven, particularly by longitudinal studies. Kokko, Tolvanen, and Pulkkinen [49] analyzed whether the level and possible changes in the level of traits and well-being across the middle adulthood are linked to each other. According to their findings, well-being is more related to the initial level of traits at age 33 than to changes in their levels. Hill, Turiano, Mroczek, and Roberts [50] came to a similar conclusion when they examined the relation between the Big Five traits and social well-being. Their findings provided support that trait development and social well-being development coincide during adulthood. Gale, Booth, Mőttus, Kuh, and Deary [51] looked at whether personality traits predict well-being in the longer term as well. They found that extraversion and neuroticisms measured in young adulthood (16–26 years) predict well-being 40 years later, when the study participants reached age 60–64.

### Personality and adaptive social functioning

Adaptive social functioning is often classified as an objective criterion of successful development in terms of comparison with the norm [23] or adaptation to social norms [52]. The criteria of adaptive social functioning include indicators such as fulfillment of developmental tasks (starting a family, finding a job, etc.), adjustment to social norms (absence of risk or antisocial behaviors, academic achievement, etc.) or medical records as objective measures of health [22].

Social functioning builds on previous human development [53–55]. According to some authors, adaptive social functioning is more firmly rooted in development than psychological functioning, which may be more dependent on actual experience [29]. Work is one of the major areas of adult life in all societies, especially career stability.

Career stability research shows that besides the influence of the family and motivation [53, 56, 57], personality traits play a substantial role as well. At present, there are a large number of studies dealing with the relation between career and adult personality. Relations between personality and subjective and objective career success were demonstrated (job satisfaction on the one hand and employment status or salary on the other) throughout adulthood [26, 58], between low neuroticism, high emotional regulation and stable career [59] or between high sense of coherence and stable career [60]. Other studies point to the relation between career stability and high agreeableness [61] and high extraversion, especially in women [59]. An unstable career, especially in the context of long-term unemployment, is associated with low subjective satisfaction and self-esteem [27, 52], high neuroticism [52], aggression [59] and higher openness to experience [61, 62].

### Current study

The current knowledge about the relationships between personality traits and well-being and adaptive functioning is mostly based on correlation studies, which do not provide information on the causality of the relationships. However, relations between personality and adaptation are bidirectional [63] and personality traits can influence life's events and be influenced by them [64]. For example, characteristics associated with unstable careers and long-term unemployment—low self-esteem or aggression—can be both a cause and a consequence of this life situation [27, 65, 66].

Previous research has also not taken into account the life-span perspective, while existing longitudinal studies have tended to focus either on prediction of adaptive functioning during childhood and adolescence [67] or during adulthood, i.e. from adolescence or young adulthood to old age [51]. However, an increasing number of studies deal with the relationship between the child's personality, temperament traits or behavioral styles and not only adult personality, but also adaptive (psychological and social) functioning in adulthood [68–72].

Shiner and Caspi [63] provided a conceptual framework for the role of childhood temperament traits in personality development and in shaping of adaptive functioning. According to their model, temperament traits that appear in early childhood before the development of other aspects of personality significantly influence children's experience of the world and shape the personality of the child, i.e. personality traits, characteristic adaptations (e.g. mental representations, typical motivations) and personal narratives [73].

Temperament widely and pervasively affects the individual’s experience as well as his or her interaction with the environment namely by means of learning process, environmental elicitation, environmental construal and environmental selection and manipulation [63]. Individual differences in temperament influence learning mechanisms such as the child’s sensitivity to positive and negative reinforcement, punishment, discrimination learning and extinction. Rothbart [74] believes that temperament provides a “meaning structure” of experience before the language develops. Whether the child will experience an event as positive or negative therefore depends on his or her emotional dispositions. Individual differences in temperament further elicit different environmental responses and affect the way other people react to the child [75]. As soon as higher cognitive functions emerge and cognitive structures develop (i.e. system of beliefs and expectations, self-concept, self-regulation mechanisms), individual differences in temperament start to gradually influence the ways children interpret (construct) their experiences with the environment, select such environment, modify it and handle it in the manner that corresponds with their personality [76]. Why temperament traits should so widely influence individual experience and environmental interactions can be explained by the fact that they are elaborated forms of basic behavioral systems such as behavioral inhibition system (BIS), behavioral activation system (BAS) or fight/flight system (FFS) [77–80].

In adulthood, personality traits remain to play an important role in influencing well-being and adaptive functioning. Similarly as in childhood, this influence is direct (emotional responses) and indirect (environmental elicitation, or selection and modification of the environment) [3, 4, 48]. Traits such as extraversion and neuroticism usually influence well-being directly by means of their emotional components (positive affectivity, negative affectivity). The indirect influence (or instrumental, as it is sometimes termed) is based on the fact that the personality traits are the source of behavior which, in the end, leads to life satisfaction. In extraverts, whose significant behavioral characteristics include sociability, well-being may be caused by positive feedback associated with a larger number of social contacts [43]. In general, personality traits are involved in many behaviors and outcomes that ensure adaptive functioning (quality of relationships, community involvement and occupational choice, satisfaction and performance) and thus contribute to well-being [81].

Today, many authors believe that the temperament is not invariable, but that it develops over time [82]. Temperament is affected mainly by parenting [75] and, subsequently, by experience and life events [64]. However, it maintains certain continuity with personality traits, both in childhood [83] and adulthood [84]. The temperament traits and later personality traits further influence how an individual adapts to his/her environment. We can therefore assume that personality characteristics from childhood and adolescence will be related to adult well-being indicators and adaptive social functioning, although these relationships are likely to be modest.

The primary objective of this study was therefore to determine whether personality traits predict well-being and stability of career in a lifelong perspective. The study also aimed to enrich existing longitudinal studies on the relationship between personality and good adaptation in two ways: 1) it dealt with the prediction of successful psychological functioning (well-being) and successful social functioning (career stability), 2) in prediction of adaptive psychological and social functioning, it took into consideration not only the personality traits from adolescence, but also child temperament from the toddler period.

As an indicator of adaptive social functioning, we used career stability, because work is an important part of life for people in middle adulthood [13, 85, 86]. Among the well-being indicators beside life satisfaction, we focused on the self-concept variables, self-esteem and self-efficacy, which is an important part of adaptive psychological functioning [38]. Like life satisfaction, self-esteem shows high stability over time [87, 88] and similar relations to personality [89, 90]. Similar relations were found between personality and self-efficacy [91–93].

## Method

### Sample

The original longitudinal study titled ‘‘The psychological development of school children coming from different social environments” was carried out by The Institute of Psychology of the Academy of Sciences of the Czech Republic between 1961 and 1980. In the beginning, the study comprised 557 children born between 1961 and 1964, the ratio of boys and girls was equal. During the longitudinal study, the dataset suffered from attrition as expected. The missing value pattern analysis revealed the anticipated regularity—the data concerning the subjects involved are usually complete until their leaving the research for different reasons. Thus, if there is a data missing concerning an individual at a certain age level, there is a high probability that there are no further details available regarding this individual. Out of the former number of 557 subjects, 331 were examined at the age of 16 (49.8% of girls). The decrease of subjects at the age of 16 years was caused by the transition from primary school to secondary school.

In 2001, the project was reactivated and attempts were made to find the original participants. A subset of the original participants was found (N = 332) and was asked to co-operate in the follow-up study focused on the life span human development. Our request letter was answered by 142 persons: 138 persons agreed to participate (54 men and 84 women), whereas three women and one man declined to co-operate. In the end, the meeting in the Institute of Psychology was attended by 83 persons (mean age at first contact, 39.7 years 48 women) between 2001 and 2005: 33 of respondents were university graduates, 39 completed secondary education and 11 finished professional school; 52 persons were married for the first time, six were unmarried, 12 were divorced, eight married again after divorce, four in another form of cohabitation after divorce, one woman married again after becoming a widow; seven persons remained childless, 21 had one child, 39 had two children, 14 had three children, one participant had 4 children, and one participant had five children (average = 1.81). The Life History Calendar method was administered in a separate session. This fact is reflected in the different number of respondents (N = 74, 41 women) in the corresponding analyses and different mean age of respondents (42.42 years). The second wave of investigations within the adulthood was done ​​in 2011, when 76 people participated in the study (44 women, mean age 48.1 years). The amount of missing data for each method followed in adulthood did not exceed the limit of 5%; missing scores were replaced using the Expectation-Maximization method, always based on other available data in the survey wave. In both waves, missing answers can be considered as missing completely at random (Little's MCAR test for wave 1: Chi-Square = 11.122, DF = 11, Sig. = 0.433, Little's MCAR test for wave 2: Chi-Square = 4.748, DF = 5, Sig. = 0.447). Due to the variable number of persons for which complete data were available for the corresponding longitudinal analysis, the results of these analyses were complemented by the effective size of the analyzed sample.

### Ethics Statement

The present study was approved by the Institutional Board of the Institute of Psychology of Academy of Sciences of the Czech Republic and written consent was obtained from participants before commencing both stages of the longitudinal study in adulthood.

### Instruments

#### Childhood

To assess children's temperament, we used examiner’s ratings of various aspects of children’s behavior during the examination. The rating scales had a 5-point response format reflecting the intensity of particular behavior. We selected twelve scales and computed their individual mean values across ages 12, 18, 24 and 30 months in the toddler period. Specifically, the following scales were used: interest in examination (m = 2.78, sd = 0.55), nervousness/neuroticism (m = 2.50, sd = 0.64), positive emotional expressions (m = 2.50, sd = 0.48), negative emotional expressions (m = 1.68, sd = 0.51), frequency of positive social responses (m = 2.48, sd = 0.52), intensity of positive social responses (m = 2.58, sd = 0.64), frequency of negative social responses (m = 1.62, sd = 0.54), intensity of negative social responses (m = 1.59, sd = 0.52), general reactivity (m = 3.01, sd = 0.59), general activity (m = 3.19, sd = 0.66), aggression against things/objects (m = 1.67, sd = 0.59), conformity/obedience (m = 3.08, sd = 0.54). The values were computed on sample N = 386. Using the factor analysis (for details of the procedure see Blatný, Jelínek, Osecká [84]) three scores reflecting the temperament traits of positive affectivity, negative affectivity and disinhibition were obtained.

#### Adolescence

At age 16, personality characteristics were measured using the Maudsley Personality Inventory [94]. Reliability in terms of internal consistency for extraversion and neuroticism scales found in our sample is satisfactory (Cronbach`s α_E_ = 0.675; Cronbach`s α_N_ = 0.857).

#### Adulthood

In both waves of data collection in adulthood, the same set of measures was administered: Eysenck Personality Inventory, NEO-FFI questionnaire, Satisfaction With Life Scale, Rosenberg Self-Esteem Scale, Generalized Self-Efficacy Scale and Life History Calendar.

Eysenck Personality Inventory [95] (EPI, Czech version by Vonkomer and Miglierini [96]) measures two dimensions—extraversion and neuroticism. Each dimension has 24 items with yes/no response format. The inventory is validated for the Czech population. Internal consistency in the first wave (Cronbach’s alpha) was 0.73 for extraversion, 0.77 for neuroticism.

NEO-FFI questionnaire [97] (Czech version by Hřebíčková and Urbánek [98]) measures five personality dimensions: extraversion, neuroticism, openness to experience, agreeableness and conscientiousness. Each dimension has 12 items with 5-point Likert scale response format. The questionnaire is validated for the Czech population. Internal consistency in the first wave was 0.85 for extraversion, 0.84 for neuroticism, 0.55 for openness, 0.70 for agreeableness and 0.80 for conscientiousness.

Satisfaction With Life Scale [99] measures overall life satisfaction. It consists of five statements with response scale expressing the degree of agreement on a scale from 1 to 5 (in the second wave of the survey, a fine-grained 9 degree response scale was used). Internal consistency in the first wave was 0.82. Rosenberg Self-Esteem Scale [100] consists of ten statements with the response scale showing the degree of agreement on a scale from 1 to 4. Cronbach’s alpha in the first wave was 0.79. Generalized Self-Efficacy Scale [101] consists of ten statements with response scale showing the degree of agreement on a scale from 1 to 4. Cronbach’s alpha in the first wave was 0.89.

Life History Calendar is a method focused on objective life events (Caspi et al., [102], modified by L. Pulkkinen, University of Jyväskylä, Finland). The method made it possible to obtain retrospective information about important events from the respondent’s life and to structure his or her autobiographical memories [103]. Two dimensions were taken into account when identifying significant life events: time (chronological) and thematic (parallel) level. Horizontal axis demonstrated time intervals (units) during which life events were recorded (in the present study, one year was established as a basic time unit—events were recorded from the 15^th^ year of age onwards). Individual domains of human life to be studied were plotted vertically [103, 104]. We focused on the following domains: living arrangements, partnership, parenthood, occupation and non-normative events (accidents, bereavement etc.). Using these data, we created a variable characterizing the respondents in terms of lifelong course of career line. According to the Finnish methodology, the career line was characterized as stable, unstable or changeable [59]. Like Rönka, Kinnunen and Pulkkinen [105], we evaluated the stability of a career from 27 years of age, to better compare people with different levels of education. A stable career is characterized by a job in the same field without periods of unemployment. The data published by the Czech Statistical Office in 2008 [106] indicate that the inhabitants of the Czech Republic spend on average 5–6 years working for the same employer without interruptions. For a stable career, the minimum duration of work for the same employer was therefore set to 5 years. Changeable careers occur among people who interrupted their careers to study or left to take care of a household (care for children, parents, etc.). If they work in the same field, they often change jobs (less than 5 years of work for the same employer). An unstable career is characterized by high variability of jobs (less than 5 years of work for the same employer) together with employment outside the field of attained education. In this group, periods of unemployment longer than 6 months can occur (not associated with waiting to be employed on a new job).

### Methods of analysis

Relations between dimensions of temperament in toddlerhood, personality traits in adolescence and adulthood and aspects of well-being in adulthood (life satisfaction, self-esteem and self-efficacy) were described by correlation analysis. A more detailed analysis of predictors of well-being in adulthood was performed using sequential regression analysis. To determine differences between groups of persons defined according to the course of their professional career in aspects of well-being in adulthood we used analysis of variance. To predict the course of a professional career on the basis of temperament dimensions in toddlerhood and of the personality traits in adolescence we used Multinomial logistic regression analysis. In the context of regression analysis, we used the bootstrapping method (1000 samples, percentile based confidence interval).

## Results

In the first step of the data analysis, we analyzed relationships between aspects of well-being and typology based on the course of career. Table 1 summarizes comparisons of groups of persons defined by the nature of their careers in self-esteem, life satisfaction and self-efficacy in the both waves of the survey. For simplicity we will hereafter use the first wave designation of *at age 40*, the second wave designation of *at age 50*.

**Table 1:** Comparison of groups of persons defined according to the course of a career in the aspects of well-being at age 40 (wave 1) and 50 (wave 2).

		Career—mean (sd)			
	wave	unstable	changeable	stable	F / partial eta squared
**self-esteem**	1	31.72 (5.57)	33.82 (3.38)	33.70 (3.28)	1.951 / 0.052
	2	31.89 (5.19)	34.65 (4.34)	33.76 (4.67)	2.100 / 0.054
**life satisfaction**	1	16.04 (4.40)^a^	18.97 (2.73)^b^	19.35 (3.03) ^b^	5.806** / 0.141
	2	28.29 (8.33) ^a^	34.96 (6.18) ^b^	31.83 (6.86) ^a^ ^,^ ^b^	5.088** / 0.122
**self-efficacy**	1	27.87 (5.68)	29.76 (5.44)	30.35 (3.80)	1.496 / 0.040
	2	29.67 (5.59)	30.78 (4.00)	29.97 (4.40)	0.490 / 0.013

** 0.01 level of significance.

Wave 1: F(2,71); N_unstable_ = 25; N_changeable_ = 29; N_stable_ = 20; wave 2: F(2,73); N_unstable_ = 24; N_changeable_ = 23; N_stable_ = 29.

^a, b^ The same upper index letter designates groups, which do not differ from each other, the different letters designates statistically different groups (based on Tukey's post hoc tests).

In cross-sectional analysis of the relations between the aspects of well-being and career course at age 40 we found significant differences in life satisfaction. Respondents with unstable careers show the lowest life satisfaction. Based on Tukey's post-hoc tests, it was found that these persons differ from people with stable careers and from people with changeable careers alike. As at 40 years of age, at age 50 there are also significant differences between groups of persons defined according to the course of their career only in life satisfaction. When specifying differences using post-hoc tests, it was found that people with unstable careers differ significantly (lowest life satisfaction) from people with changeable careers (highest life satisfaction).

The next stage of data analysis cross-sectionally investigated relations between personality characteristics and aspects of well-being using correlation analysis. Table 2 lists the values of correlation coefficients for age 40 and the values for age 50.

**Table 2:** Relations between personality dimensions and aspects of well-being at age 40 and 50.

	wave	self-esteem	life satisfaction	self-efficacy
**extraversion (EPI)**	1	0.238*	0.206	0.291**
	2	0.150	0.166	0.253*
**neuroticism (EPI)**	1	-0.505**	-0.368**	-0.368**
	2	-0.531**	-0.369**	-0.427**
**neuroticism (NEO-FFI)**	1	-0.629**	-0.436**	-0.510**
	2	-0.715**	-0.564**	-0.631**
**extraversion (NEO-FFI)**	1	0.406**	0.321**	0.429**
	2	0.396**	0.414**	0.527**
**openness (NEO-FFI)**	1	0.127	0.142	0.182
	2	-0.070	-0.103	0.050
**agreeableness (NEO-FFI)**	1	0.153	0.173	-0.173
	2	0.348**	0.257*	0.235*
**conscientiousness (NEO-FFI)**	1	0.431**	0.187	0.479**
	2	0.489**	0.245*	0.498**

* Correlation is significant at the 0.05 level;

** correlation is significant at the 0.01 level.

On the basis of correlation analysis, we can conclude that with the exception of openness to experience, personality traits are relatively closely linked to the characteristics of well-being. From the basic personality traits measured by the NEO-FFI questionnaire, the traits of neuroticism, extraversion and conscientiousness are most associated with well-being. The relations found are thus consistent with the findings of other studies on the relation between personality and well-being.

In the main part of the analysis, we focused on prediction of aspects of well-being in adulthood using data on the characteristics of temperament in toddlerhood and personality traits in adolescence. Table 3 shows the values of the corresponding correlation coefficients.

**Table 3:** Relations between temperament in toddlerhood (1–3 y.), personality in adolescence (16 y.) and aspects of well-being at age 40 (N = 69) and 50 (N = 64).

	40 years (N = 69)			50 years (N = 64)		
	self-esteem	life satisfaction	self-efficacy	self-esteem	life satisfaction	self-efficacy
**positive affectivity**	0.046	0.197	0.070	0.070	0.143	0.053
**disinhibition**	0.097	0.060	0.326**	0.097	0.120	0.279*
**negative affectivity**	0.058	-0.049	0.188	-0.070	-0.035	0.056
**extraversion**	0.339**	0.393**	0.351**	0.320**	0.272*	0.347**
**neuroticism**	-0.089	-0.162	-0.200	-0.222	-0.154	-0.267*

* Correlation is significant at the 0.05 level;

** correlation is significant at the 0.01 level.

Table 3 shows that the level of aspects of well-being in adulthood are associated in particular with extraversion in adolescence, with neuroticism in the same period and level of disinhibition observed in toddlerhood. To clarify the role of temperament and personality characteristics in predicting the characteristics of individual well-being in adulthood, we used the method of sequential regression analysis. In the first block children's temperament dimensions were entered as predictors (positive affectivity, disinhibition, negative affectivity), in the second block we used variables capturing personality in adolescence (extraversion, neuroticism).

Of the three studied early temperament characteristics only the dimension of disinhibition allows the prediction of well-being (model 1 in Tables 4 and 5). Specifically, we found a statistically significant regression coefficient for the relation between child disinhibition and self-efficacy at the age of 40. At the age of 50, this relation was close to 5% level of significance (p = 0.054). If we focus on the results of model 2 (with predictors from block 1 and block 2), we can conclude that the addition of the block of personality variables collected during adolescence almost always resulted in a statistically significant increase in the explained variance of predicted indicators of well-being (the only exception is life satisfaction at age 50). The increase in the explained variance can be attributed almost uniquely to extraversion.

**Table 4:** Prediction of well-being at age 40 based on the characteristics of temperament in toddlerhood (1–3 y.) and personality characteristics in adolescence (16 y.).

		self-esteem	life satisfaction	self-efficacy
**model 1**	**positive affectivity (β)**	0.033	0.209	-0.011
	**disinhibition (β)**	0.072	-0.020	0.302*
	**negative affectivity (β)**	0.047	0.015	0.102
	**R** ^**2**^	0.011	0.039	0.117*
**model 2**	**positive affectivity (β)**	-0.049	0.119	-0.079
	**disinhibition (β)**	0.089	-0.001	0.316*
	**negative affectivity (β)**	-0.064	-0.121	-0.006
	**extraversion (β)**	0.385**	0.410*	0.301
	**neuroticism (β)**	0.079	-0.003	-0.047
	**ΔR** ^**2**^	0.115*	0.152**	0.095*
	**R** ^**2**^	0.126	0.191*	0.212*

* 0.05 level of significance;

** 0.01 level of significance.

The table shows standardized regression coefficients for individual predictors.

**Table 5:** Prediction of well-being at age 50 based on the characteristics of temperament in toddlerhood (1–3 y.) and personality characteristics in adolescence (16 y.).

		self-esteem	life satisfaction	self-efficacy
**model 1**	**positive affectivity (β)**	-0.013	0.096	-0.069
	**disinhibition (β)**	0.155	0.115	0.336
	**negative affectivity (β)**	-0.135	-0.052	-0.098
	**R** ^**2**^	0.024	0.029	0.085
**model 2**	**positive affectivity**	-0.059	0.059	-0.112
	**disinhibition (β)**	0.125	0.094	0.306
	**negative affectivity (β)**	-0.220	-0.118	-00.18
	**extraversion (β)**	0.307*	0.260	0.280
	**neuroticism (β)**	-0.089	-0.030	-0.115
	**ΔR** ^**2**^	0.118*	0.070	0.113*
	**R** ^**2**^	0.141	0.099	0.198*

* 0.05 level of significance;

** 0.01 level of significance.

The table shows standardized regression coefficients for individual predictors.

The next step of the analysis monitored the relation between dimensions of toddler temperament, personality characteristics in adolescence and typology based on career characteristics at age 40 and 50. At age 40, respective data was available from 22 respondents with unstable careers, 24 respondents with changeable careers and 14 respondents with stable careers. At the age of 50 years, the number of persons with unstable careers was 22, 18 with changeable and 24 with stable careers.

Although using Multinomial logistic regression we found that the dimensions of children's temperament and personality traits in adolescence allow the prediction of the career course at age 40 (χ^2^ = 19.188; df = 10; p = 0.038; Cox and Snell pseudo R^2^ = 0,274; Nagelkerke R^2^ = 0.310), no predictor showed any statistically significant independent effect on overall career course (positive affectivity: χ^2^ = 1.191; df = 2; p = 0.551; disinhibition: χ^2^ = 3.663; df = 2; p = 0.160; negative affectivity: χ^2^ = 5.259; df = 2; p = 0.072; extraversion: χ^2^ = 4.739; df = 2; p = 0.094; neuroticism: χ^2^ = 0.355; df = 2; p = 0.837).

Dimensions of children's temperament and personality traits in adolescence allow prediction of the career course at age 50 (χ^2^ = 22.328; df = 10; p = 0.014; Cox and Snell pseudo R^2^ = 0.295; Nagelkerke R^2^ = 0.332). In this case, the significant predictors include disinhibition, negative affectivity and extraversion (see Table 6).

**Table 6:** Prediction of professional career type at age 50 based on the characteristics of temperament in toddlerhood (1–3 y.) and personality characteristics in adolescence (16 y.).

predictor	χ^2^(2)	significant paired comparisons (p < 0.05)	B(SE)	exp(B)	95% confidence interval
**positive affectivity**	0.417				
**disinhibition**	7.444*	stable vs. unstable	1.606 (0.813)	4.984	(1.800; 45.971)
		changeable vs. unstable	1.271 (0.937)	3.565	(1.046; 36.598)
**negative affectivity**	10.884**	stable vs. unstable	-1.552 (0.761)	0.212	(0.028; 0.513)
		changeable vs. unstable	-1.223 (0.762)	0.294	(0.042; 0.787)
**extraversion**	7.760*	changeable vs. unstable	0.155 (0.137)	1.168	(1.055; 1.504)
**neuroticism**	1.523				

* 0.05 level of significance;

** 0.01 level of significance.

It is evident that disinhibition and negative affectivity play an important role in the prediction of professional career type. Based on the size of odds ratios we can conclude that their influence is of similar magnitude. More specifically, higher level of disinhibition increases the likelihood of a stable career in comparison with an unstable career; with higher level of negative affectivity, on the contrary, the likelihood of a stable career decreases in comparison with an unstable career. Higher level of disinhibition also increases the likelihood of changeable career, compared with an unstable career, and higher level of negative affectivity decreases the likelihood of changeable career compared with unstable career. It was also found that higher level of extraversion increases the likelihood of the career being changeable, compared to unstable.

## Discussion

In this study, we used longitudinal data to examine whether it is possible to predict adaptive psychological and social functioning in adulthood on the basis of temperament from toddlerhood and personality traits from adolescence. We found that 1) the child's temperament and personality from adolescence predict both adaptive psychological functioning, well-being (life satisfaction, self-esteem, self-efficacy) and adaptive social functioning (career stability), 2) more specifically, extraversion from the age of 16 and toddler temperament dimension of disinhibition apply in the prediction of well-being and career stability; an unstable career is further predicted by negative affectivity in childhood.

To describe the child's temperament, we used the assessment of children's behavior by an examiner during regular psychological examinations [84]. The study initiated in 1961 (in fact in 1960, because the parents were contacted before the birth of a child) did not include the standard method for diagnosing child temperament. The scales we used could thus influence the identified factor structure of child temperament. However, the dimensions found—positive affectivity, negative affectivity and disinhibition—corresponded with e.g. Clark's [107] general structure of temperament based on the review of temperament and personality psychopathology researches.

The positive affectivity factor (positive social responses, positive emotional expressions and interest in examination) and the negative affectivity factor (negative social responses, negative emotional expressions and nervousness) can be clearly interpreted. The factor referred to as disinhibition covers a scale of aggression against things/objects, general activity, low conformity/obedience, and general reactivity and can therefore be interpreted as an assertion/activity factor. We must continue to take this into account when interpreting the results of relations between children's temperament and psychological characteristics and social functioning in adulthood.

As to the results of this study, we first examined the relationship between adaptive psychological and social functioning in adulthood. We found, as in other studies, that adaptive psychological and social functioning are related: the lowest level of life satisfaction was found in people with an unstable career. Also, self-esteem and self-efficacy were the lowest among people with an unstable career, as expected, although these differences did not prove to be statistically significant. The relationship between well-being and social functioning, namely career stability, has been confirmed repeatedly: an unstable career associated with unemployment significantly reduces the level of life satisfaction [27, 108, 109]. On the other hand, career success is a significant predictor of well-being. A stable career, job satisfaction, good relationships in the workplace and reasonable income increase the level of well-being [28, 110]. In certain cultural and social conditions, differences between the unemployed (people with unstable career) and the employed do not manifest themselves at all [111], especially where unemployment (unstable career) has become the norm in society [112].

The main objective of this study was to explore the relationships between adaptive psychological and social functioning in adulthood and temperamental and personality characteristics in childhood and adolescence. Regarding adaptive psychological functioning, relationships between aspects of well-being and personality traits in adulthood show usual pattern of relationships, in which neuroticism correlates most negatively with life satisfaction, self-esteem, and self-efficacy, whereas extraversion and conscientiousness correlate positively [113, 114]. There is only one difference between the ages of 40 and 50: it is the relationship between aspects of well-being and agreeableness, which does not correlate with well-being at the age of 40, while at the age of 50 it does. Kokko et al. [49] came to similar results—they found, based on longitudinal data with the same persons, that agreeableness does not correlate with life satisfaction at age 36 and 42, while at 50, it does. Similarly, in a cross sectional study with adolescents and middle and late adults Butkovic, Brkovic, and Bratko [115] found that agreeableness was linked to well-being in the older cohort, but not in adolescents. Although the literature provides insufficient sources for reliable interpretation of these findings, Shallcross, Ford, Floerke, and Mauss [116] observed that feelings of anger and anxiety decrease with increasing age and that increasing age is associated with increased acceptance of negative emotional experiences and this process mediates the relationship between age on the one hand and anger and anxiety on the other hand. In the five factor model of personality, anger/hostility is a component of neuroticism which, however, correlates with several facets as well with a total agreeableness score [97, 98]. Changes in negative emotions and their acceptance therefore can relate to changes in relationships between agreeableness and emotional well-being.

However, from a longitudinal perspective, neuroticism is not the best predictor of aspects of well-being in adulthood (it is not involved in prediction at all), it is extraversion: extraversion at 16 years of age predicts self-esteem and life satisfaction at 40 and self-esteem at 50. Taking into account the correlation analysis results, extraversion from adolescence is related to all aspects of well-being at both ages in adulthood, whereas neuroticism correlates (negatively) only with self-efficacy at 50. This observation is rather surprising, as for example Gale et al. [51] found that mental well-being and life satisfaction at age 60–64 are predicted by data on the level of extraversion and neuroticism for the period of 16 to 26 years of age. On the other hand, our results are supported by a study by Gomez, Krings, Bangerter, and Grob [117], who found on the basis of cross-sectional data from three age cohorts (young adults—average age 26 years, middle-aged adults—51 years and old adults—76 years) that extraversion is only a predictor of SWB in young adults and the effect of neuroticism is more pronounced in old adults. Moreover, non-significant correlations between neuroticism and well-being identified in the present study cannot be interpreted as the absence of the relationships due to the relatively small number of respondents. The correlation between neuroticism in adolescence and aspects of well-being in adulthood range from -0.089 to -0.267 (median correlation = -0.211), and with a larger sample, the relationships might prove significant.

Temperament in toddlerhood also applies in the prediction of adult adaptive psychological functioning, namely the disinhibition dimension predicts self-efficacy at age 40 and 50 (at 50, this relationship was close to 5% level of significance, p = 0.054). Research with which we could compare our results is scarce. However, our results correspond with the work of Caspi and colleagues [118, 119] who investigated the relationship between children's early-emerging behavioral styles at 3 years of age and their characteristic behaviors, thoughts, and feelings as adolescents and adults. Children diagnosed as inhibited had, in comparison with other types, in adolescence and young adulthood (18–26 years old) the highest trait levels of constraint (particularly harm avoidance) and the lowest trait levels of positive emotionality (particularly social potency, achievement, and well-being), were assessed by others as low affectionate, outgoing and vital, and were significantly more likely to be diagnosed with depression. Further comparison with Caspi´s research is difficult because Caspi and colleagues used typological approach to temperament and diagnosed behavioral styles at age 3 and therefore more characteristics were included, including those that reflected self-consciousness.

However, not only well-being, but also social functioning and career stability can be predicted from the personality traits of childhood and adolescence. At age 50, a higher level of extraversion increases the likelihood that the individual's career will be changeable compared to unstable; career stability is further predicted by child disinhibition and negative affectivity: a higher level of disinhibition increases the likelihood of a stable and changeable career compared to an unstable career, while higher level of negative affectivity decreases the likelihood of a stable and changeable career compared to an unstable career. So far, most studies have examined the relationship between personality and career in adulthood and found a relationship between stable career and low neuroticism, high extraversion and agreeableness [59, 61]. Our results thus support the existence of the relationship between extraversion and career stability also from the longitudinal point of view. The absence of significant relationship between neuroticism and occupational aspects of social functioning corresponds with a weak relationship identified between neuroticism and aspects of psychological functioning in adulthood (well-being).

Our research has shown that a stable career is associated with disinhibited temperament in early childhood (assertiveness, activity, low conformity, general reactivity) and an unstable career is associated with negative affectivity (negative social responses, negative emotional expressions and high nervousness). Similarly, other longitudinal studies on the prediction of social (career) functioning have found an association with early temperament. Caspi and colleagues [120, 121] found that well-adjusted children had good results in the area of work and under-controlled children had poor work social interactions. In particular characteristics of under-controlled children are very similar to our group of children with high negative affectivity: they are negativistic, very irritable with emotionally labile responses.

Unlike other studies that link extraversion to stable career [122], our results rather point to the relationship between extraversion and changeable career. Changeable career occurred more frequently in our sample than in other studies [27]. This result can be explained on the basis of macro-social changes that occurred in the Czech Republic at the time when the careers of the longitudinal study participants were in their initial stages.

In 1989, the so-called Velvet Revolution took place in the former Czechoslovakia, which led to the dissolution of the totalitarian communist regime and the transition to a democratic society, with the dissolution of Czechoslovakia following shortly afterwards in 1993. At the time of this study, the participants were in their young adulthood—in the period of life when people set up their own families, enter the job market and therefore are much more sensitive to changes taking place in their social environment than in other developmental stages [123]. The Velvet Revolution led to significant macro-social changes and a relatively stable society was transformed into a transitional society. In the field of employment—in common with other post-communist countries (Poland, Hungary)—this change manifested itself by a phenomenon affecting career stability that was previously virtually non-existent: unemployment [124, 125], which started to increase mainly in young people [126]. The economic system of the former Czechoslovakia underwent major changes such as transformation of industry, closure of heavy manufacturing, privatization of state-owned enterprises etc. These changes significantly contributed to the diversification of career trajectories that used to be rather uniform before 1989 [127]. According to previous research [111, 128], macro-social changes first lead to changes in social functioning characteristics. The 1989 events in Czechoslovakia brought about significant and sudden positive changes in areas such as education or travel (free choice of study, free travel abroad), but also shook the existing “security” in relation to jobs [129, 130]. The macro-social change therefore may not always be perceived as only positive or only negative. Depending on the circumstances, people can perceive the new demands of society as a challenge or as a threat [131]. In the field of employment, especially in comparison with the previous and upcoming historical period, people got a chance to experiment more—to establish their own business, work abroad and try new, unusual or previously untested professions [124]. Some longitudinal studies focusing on the lifelong course of career of young people living in post-communist countries describe the so called “cohort of winners” [132]. These people were 20 to 30 years old at the time of political transition. As they were young and had just started their career, they were more inclined than older workers to change their career direction or to take entrepreneurial opportunities [133].

In predicting both adaptive psychological and adaptive social functioning, extraversion measured at 16 years of age and the dimension of child disinhibition are therefore mainly applied. Common components of extraversion and child disinhibition are activity and assertiveness. So it seems that characteristics such as activity, vigor and assertiveness are more important at the beginning of adulthood for good future adjustment than emotional stability or agreeableness, which becomes increasingly important in later life. This composition of traits could be interpreted as a kind of viability/vitality which could be viewed as a tendency towards a positive approach to life and active adaptation to life conditions.

Even though we had only the traits of extraversion and neuroticism available in our research in adolescence, the concept of higher order traits of the Big Five personality constructs is becoming an ever more appropriate interpretative framework for the interpretation of our results. Digman [134] found out the existence of higher-order factors, which he referred to as alpha and beta. Alpha includes emotional stability, conscientiousness and agreeableness and reflects the process of socialization and expresses the relative ability to control one’s behavior. Beta includes extraversion and openness to experience and expresses the characteristic of personal growth. DeYoung [135, 136] proposed referring to higher order factors as stability (emotional stability, conscientiousness and agreeableness) and plasticity (extraversion and openness to experience). Whereas Digman conceived of the higher order factors rather as life outcomes, De Young suggested re-conceptualizing the stability dimension as reflecting individual differences in the basic tendency of human beings to maintain a stable constitutional organization and the plasticity dimension as reflecting individual differences in the basic tendency to incorporate novel information into that organization.

Subsequent research has verified the existence of higher-order factors [137–139] and showed the expected distinct relationships to other variables—to conformity [135], externalized psychopathology [140], engagement and restrain [141], threat and exploration narratives [142], job performance [143] and mental health [139]. Plasticity is associated with low conformity, externalized psychopathology, engagement, exploration and PWB (specifically the facets of personal growth and autonomy), whereas stability is associated with conformity, restrain, absence of threat narratives and life satisfaction.

Wang, Chen, Petrill, and Deater-Deckard [144] and Slobodskaya [145] identified the traits of stability and plasticity in children and adolescents aged 3.5 to 12 years and 3 to 17 years. In addition, Wang's et al. cross-sectional analyses indicated higher plasticity among younger children and higher stability among older children. It seems, therefore, that the stability and plasticity factors may play different roles at different stages of development. Research on the development of meta-traits is scarce, but we can build on the Big Five investigation: the levels of extraversion and openness to experience, i.e. the level of traits constituting plasticity, decrease in the course of life, whereas the levels of emotional stability, conscientiousness and agreeableness, i.e. the level of traits constituting stability, increase [146] (recent investigations suggest that e.g. agreeableness and conscientiousness increased among young cohorts, are stable among middle-aged cohorts and declined among the oldest cohort [147]). These changes may reflect different importance of personality traits for adaptive functioning throughout life. While active adaptation and acquisition of new information are important in young adulthood, the tendency to maintain stable relations and their optimization come to the foreground in middle adulthood [20].

Even though from adolescence we only had data on the level of extraversion and neuroticism, each of these traits represents a different meta-trait—extraversion represents plasticity and neuroticism (or emotional stability) represents stability. Our results seem to support the hypothesis that in adolescence and young adulthood, traits associated with plasticity are important for later adaptive functioning (flexibility, agency, development and personal growth), while traits associated with stability gain importance in middle adulthood (ability to maintain stable interpersonal relationships, motivational system and emotional conditions).

## Limitations

One of the main objectives of the study was to predict adaptive functioning in adulthood based on temperament traits identified in toddlerhood. As mentioned in the introduction to the discussion, temperament in childhood has not been determined using standard measurement tools, but rather derived from rating scales of children's behavior from psychological examinations. Although the found temperament structure could have been influenced by this factor, the temperament dimensions we identified correspond to theoretical assumptions.

Other limitations of the study stem from the relatively low number of subjects for whom the necessary data for analysis of hypothesized relationships were available. Although the research sample was quite large at the beginning of the longitudinal study (over 500 children), we managed to contact only a relatively small portion of the original sample in adulthood. The sample attrition rate was, apart from standard factors, definitely affected by the long time span between the end of the original research (in middle adolescence of the respondents) and the initiation of the new follow-up study in middle adulthood. The relatively low number of respondents to some extent limits the validity of the conclusions made ​​on the basis of inferential statistics procedures.

Another limiting factor is that well-being was not measured in adolescence. Consequently, we could not verify the relationship between WB and personality traits, as we did in adulthood. If we had cross-sectional data from adolescence at our disposal, we would have been able to better clarify the predictive potential of personality traits for WB in adulthood.
